# Matrin 3 is a co-factor for HIV-1 Rev in regulating post-transcriptional viral gene expression

**DOI:** 10.1186/1742-4690-8-61

**Published:** 2011-07-20

**Authors:** Venkat SRK Yedavalli, Kuan-Teh Jeang

**Affiliations:** 1 Molecular Virology Section, Laboratory of Molecular Microbiology, National Institutes of Allergy and Infectious Diseases, the National Institutes of Health, Bethesda, Maryland 20892-0460, USA

**Keywords:** Matrin 3, HIV-1, Rev, RNA export, nuclear matrix protein

## Abstract

Post-transcriptional regulation of HIV-1 gene expression is mediated by interactions between viral transcripts and viral/cellular proteins. For HIV-1, post-transcriptional nuclear control allows for the export of intron-containing RNAs which are normally retained in the nucleus. Specific signals on the viral RNAs, such as instability sequences (INS) and Rev responsive element (RRE), are binding sites for viral and cellular factors that serve to regulate RNA-export. The HIV-1 encoded viral Rev protein binds to the RRE found on unspliced and incompletely spliced viral RNAs. Binding by Rev directs the export of these RNAs from the nucleus to the cytoplasm. Previously, Rev co-factors have been found to include cellular factors such as CRM1, DDX3, PIMT and others. In this work, the nuclear matrix protein Matrin 3 is shown to bind Rev/RRE-containing viral RNA. This binding interaction stabilizes unspliced and partially spliced HIV-1 transcripts leading to increased cytoplasmic expression of these viral RNAs.

## Background

The nucleus is a highly organized structure. Chromosomes occupy discrete regions, and specific proteins and nucleic acids are enriched in subnuclear structures such as nuclear lamina, nucleoli, Cajal bodies, nuclear speckles, and paraspeckles [1-6]. The nuclear matrix, a network of underlying filaments in the cell nucleus, shapes the nuclear architecture and functions in genome maintenance, transcription and RNA metabolism [7-17]. Accordingly, the nuclear matrix has important roles in tissue development and cellular proliferation; and the disruption of nuclear organization is often correlated with disease states such as the loss of subnuclear promyelocytic leukemia bodies in acute promyelocytic leukemia [18-21].

HIV-1 gene expression and replication are regulated at transcriptional and post-transcriptional steps including the transactivation of the HIV-1 LTR by Tat [22] and the export of unspliced or partially spliced viral RNAs from the nucleus to the cytoplasm by Rev [23-26]. Rev is a trans-acting viral protein which binds to a cis-acting Rev responsive element (RRE) present in unspliced and partially spliced HIV transcripts. Rev has been shown to interact with cellular proteins CRM1, DDX3, PIMT and others to mediate the export of unspliced and singly spliced viral RNAs [27-30]. The mechanism of viral RNA export by Rev is discrete from the export pathways used by fully spliced HIV-1 mRNAs, CTE- (constitutive transport element) dependent RNAs, and cellular mRNAs [31-43].

Recently, numerous studies have implicated the nuclear matrix in gene transcription, RNA splicing, and transport of cellular RNAs [5,7,9,44,45]; however, the role of the nuclear matrix in HIV-1 gene expression has been poorly explored [46-48]. Here, we identify Matrin 3 as a key component of factors that mediate the post-transcriptional regulation of HIV-1. Matrin 3 is a highly conserved inner nuclear matrix protein which has been previously shown to play a role in transcription [49-52]. It interacts with other nuclear matrix proteins to form the internal fibrogranular network; it acts in the nuclear retention of promiscuously A-to-I edited RNAs in cooperation with p54(nrb) and PSF [53,54]; it participates in NMDA-induced neuronal death; it modulates the promoter activity of genes proximal to matrix/scaffold attachment region (MAR/SAR) [55]; and it is involved in the repair of double strand breaks [56]. Our current findings implicate that Matrin 3 also influences the post-transcriptional expression of a subset of HIV-1 mRNAs.

## Results

### Matrin 3 enhances Rev/RRE directed gene expression

We identified Matrin 3 as a PTB-1 (polypyrimidine tract binding protein -1) interacting protein in a yeast 2 hybrid assay (Table 1). PTB -1 plays a role in the alternative splicing of cellular mRNAs and has been described to promote the expression of fully spliced HIV-1 transcripts (our unpublished results and [57]). A "PTB-1 associated splicing factor" [58] named PSF has been proposed to inhibit the expression of HIV-1 unspliced/spliced transcripts [59]. We reasoned that like PSF, Matrin 3 through its association with PTB-1 might modulate HIV-1 gene expression.

**Table 1:** List of Human and Mouse PTB-1 interacting proteins identified by yeast 2 hybrid assay.

PTB-1 interacting proteins identified by yeast 2 hybrid assay	Other names/synonyms	Accession #
A) ***Interacting with Human PTB-1***		

Aladin	AAAS; adracalin	NP_056480

Calcium and integrin binding 1	CIB1; CIB; kinase-interacting protein 1; KIP1	NP_006375

Cleavage stimulation factor, 3' pre-RNA, subunit 2, 64 kD, tau	CSTF2T; KIAA0689	NP_056050

Homeodomain-interacting protein kinase 1 isoform1	HIPK1; KIAA0630	NP_938009

***Matrin 3***	***MATR3***	***NP_001181883***

poly(rC) binding protein 1	PCBP1	NP_006187

RNA binding motif protein 10	RBM10	NP_005667

Exportin 1	CRM1; XPO1	NP_003391

heterogeneous nuclear ribonucleoprotein K, isoform b	HNRPK	NP_112552

heterogeneous nuclear ribonucleoprotein L	HNRPL	NP_001524

Raver1	Raver1	NP_597709

A) ***Interacting with Mouse PTB-1***		

arylhydrocarbon receptor nuclear translocator	ARNT, hypoxia-inducible factor 1, beta subunit; dioxin receptor	NP_001659

Calcium and integrin binding 1	CIB1; CIB; KINASE-INTERACTING PROTEIN 1; KIP1	NP_006375

DAZ associated protein 2	DAZAP2	NP_055579

nuclear receptor coactivator 6	NCOA6	NP_054790

Raver1	Raver1	NP_597709

***Matrin 3***	***MATR3***	***NP_001181883***

RNA binding motif protein 10	RBM10	NP_005667

fibrosin-1-like protein	KIAA1545; FBRSL1	NP_001136113

protein BAT2-like 1	KIAA0515; BAT2L1	NP_037450

hexaribonucleotide binding protein 3	HRNBP3; RBFOX3; FOX3	NP_001076044

G protein pathway suppressor 2	GPS2	NP_004480

proline rich 3	PRR3	NP_079539

tripartite motif-containing 8	TRIM8	NP_112174

zinc finger, CCHC domain containing 2	ZCCHC2;	NP_060212

zinc finger protein 36, C3H type, homolog	ZFP36A, tristetraprolin; NUP475	NP_003398

neuro-oncological ventral antigen 1	NOVA1	NP_002506

neuro-oncological ventral antigen 2	NOVA2	NP_002507

Matrin 3 was identified to interact with both Human and Mouse PTB-1 (indicated in bold and italics. The yeast 2 hybrid screening was performed at Myriad Pronet (Utah, USA) using human and mouse PTB-1 as bait. PTB-1 interacting proteins were identified using activation domain fused libraries obtained from human spleen, brain and heart.

**Figure 1:**
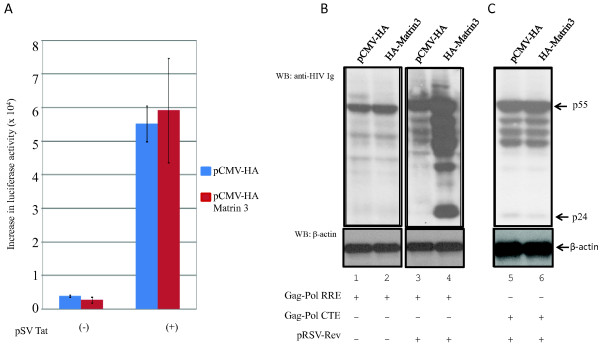
**Matrin 3 promotes the expression of Rev dependent RRE containing transcripts**. A) HeLa cells were transfected with Matrin 3 and Tat along with HIV-1 LTR luciferase. Luciferase assays performed on cell lysates prepared from these cells did not show any effect of Matrin 3 on Tat dependent LTR transactivation. B) Matrin 3 enhances the expression of RRE containing RNA transcripts in the presence of Rev in HeLa cells. HeLa cells were transfected with 2.0 μg of Matrin 3 expression or control plasmid along with 0.5 μg of pCMV -GagPol-RRE plasmids in the presence or absence of Rev. HA-Matrin 3 significantly increased the expression of Gag from the reporter construct pCMV-GagPol-RRE in the presence of Rev (compare lanes 2 and 4). C) Gag expression from CTE containing pCMV-GagPol-CTE reporter was not effected by HA-Matrin 3 (compare lanes 5 and 6).

To explore a role for Matrin 3 in HIV-1 replication, we measured the effect of over expressed Matrin 3 on viral Tat and Rev mediated gene expression. We expressed Matrin 3 and Tat, either separately or together, in HeLa cells with an HIV-1 LTR luciferase plasmid and measured reporter-expression. As shown in Figure 1A, Matrin 3 did not influence either basal LTR expression or Tat activated expression, suggesting that it does not act at the step of transcription.

We next investigated if Matrin 3 acts at steps post transcription. Rev is required for the cytoplasmic localization of unspliced and partially spliced HIV-1 mRNAs that encode for viral Gag, Env, Vif and Vpu proteins. Rev binds to an RRE-RNA motif in these RNAs [60,61]. Unlike fully spliced viral RNAs, these transcripts contain cis-inhibitory RNA elements which restrict their export from the nucleus into the cytoplasm in the absence of Rev binding to the RRE motif. The binding of Rev to the RRE frees this restriction, and Gag protein expression is thus increased by several fold compared to its expression in the absence of Rev [60,61].

We checked if Matrin 3 affects Rev-mediated post-transcriptional processes by using a CMV-promoter driven Gag-Pol-RRE expression plasmid as a reporter. HeLa cells were transfected with wild type and mutant Matrin 3 together with pCMV Gag-Pol RRE, as indicated; and 24 hours later, cells were harvested and cell lysates were analyzed by Western blotting. Figure 1B (lanes 1 and 2) shows that Matrin 3 did not alter the expression of Gag in the absence of Rev; however, in the presence of Rev, Matrin 3 increased Gag expression by approximately 10 fold (Figure 1B, lanes 3 and 4). These results support a role for Matrin 3 in Rev-dependent expression of RRE-containing HIV-1 transcripts.

The CTE is a *cis*-motif found in RNAs from simple type D retroviruses [32]. It recruits cellular RNA-binding proteins that act to export unspliced or partially spliced viral mRNAs from the nucleus into the cytoplasm [39,41]. Artificial placement of the CTE into HIV-1 Gag RNA facilitates its cytoplasmic export and expression, independent of Rev/RRE function [32]. Indeed, CTE and Rev/RRE describe two separate pathways such that the inhibition of either pathway does not affect the export of RNA through the other pathway [34,35]. We next assayed a Gag expression vector in which the RRE was replaced with a CTE. Unlike the results from Gal-Pol-RRE (Figure 1b), we found that the over expression of Matrin 3 had no effect on Gag-Pol-CTE expression (Figure 1C, lanes 5 and 6).

**Figure 2:**
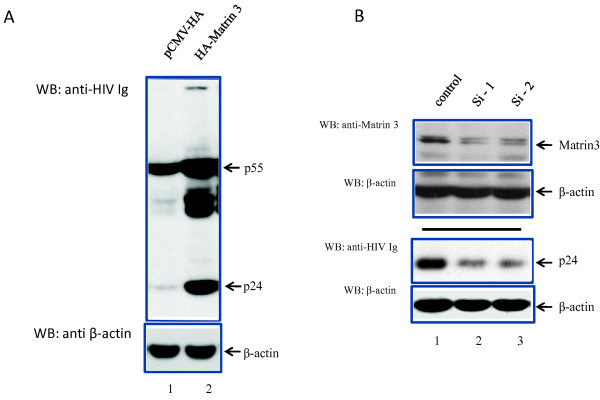
**Matrin 3 increases HIV-1 production from transiently tranfected HeLa cells**. A) HeLa cells were transfected with pNL4-3 along with WT Matrin 3, and the expression of viral proteins was analyzed on Western blots. Wild type HA-Matrin 3 (lane 2) enhanced viral protein expression. B) Matrin 3 knockdown using siRNA efficiently decreased cell endogenous Matrin 3 (lanes 2 and 3; top panel). Controls were scrambled irrelevant siRNAs. (lower two panels) HeLa cells were transfected with HIV-1 molecular clone pNL4-3 and either the control or the siRNA targeting Matrin 3. Western blot analysis of cell lysates showed that siRNA-mediated Matrin 3 knockdown reduced HIV-1 expression as indicated by decreased p24 expression (lanes 2 and 3). Loadings were normalized to β-actin.

It would be physiologically important to replicate the observations made on the Gag-Pol reporters using a full length HIV-1 infectious molecular clone, pNL4-3. We thus transfected HeLa cells with pNL4-3 and either a control vector or a Matrin 3 expressing vector. One day after transfection, cell lysates were immunoblotted for p24 Gag; and we found that Matrin 3 increased p24 Gag level by approximately 10 fold (Figure 2A). In a complementary experiment, Matrin 3 RNA was knocked down using specific siRNAs (Figure 2B). siRNA-mediated knock down of Matrin 3 decreased HIV-1 p24 Gag expression from pNL4-3 by 3 to 4 fold (Figure 2B). On the other hand, when Matrin 3 expression in knocked down cells was reconstituted (Additional file 1, Figure S1), HIV-1 gene expression was restored. Collectively, the results are consistent with Matrin 3 selectively acting on HIV-1 Rev/RRE - dependent post-transcriptional events.

### Matrin 3 interacts with Rev

**Figure 3:**
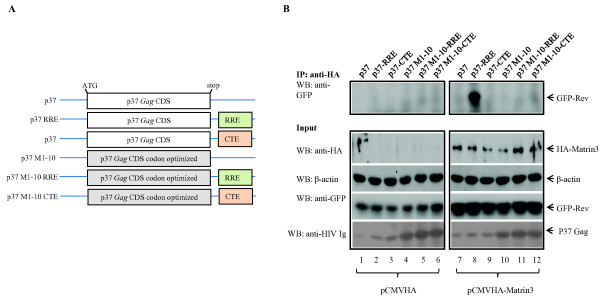
**Matrin 3 interacts with Rev in the presence of viral Rev-dependent RRE-containing RNA**. A) Schematic representations of the RNAs expressed from the various p37Gag constructs. B) Co-immunoprecipitation of GFP-Rev occurs only in the context of p37-RRE. HeLa cells were transfected with either pCMV-HA (lanes 1-6), or pCMVHA-Matrin 3 (lane 7-12) and GFP-Rev (lanes 1-12) plasmids, along with the indicated versions of a p37Gag expression construct (see panel A and as indicated). Cell lysates were subjected to immunoprecipitation with anti-HA antibody. Western blot analysis of co-immunoprecipitations shows that interaction occurs between Rev and Matrin 3 in the presence of co-transfected p37RRE (lane 8, top panel) construct, but not p37, p37CTE, or codon optimized P37 Gag constructs that are Rev-independent (lanes 7 and 9-12, top panel). Lower two panels show the expression of Rev and Matrin 3 in cell lysates used for the immunoprecipitations, and the second panel from the top shows HA-Matrin 3 proteins recovered by the co-immunoprecipitations.

How does Matrin 3 affect Rev/RRE-dependent expression? We wondered if Rev, Matrin 3 and RRE-containing RNA are together in a ribonucleoprotein complex. To check this possibility, we transfected and immunoprecipitated HeLa cells with EGFP-Rev with or without Matrin 3 along with versions of HIV-1 Gag p37 constructs (Figure 3A) with or without RRE or CTE [62-64]. The immunoprecipitates were then analyzed by Western blotting using either anti-HA or anti-GFP. Figure 3 shows that there was no interaction between Rev and Matrin 3 (Figure 3B, lanes 7, 9, 10, 11, 12), except when a p37-RRE plasmid was expressed (p37RRE; Figure 3B, lane 8; top). This interaction was not seen when a p37CTE plasmid was used in place of p37RRE (Figure 3B, lanes 9) or when the p37 Gag sequences were codon optimized to make the expression of the RNA transcripts Rev-independent (Figure 3B, lanes 10-12) [62-64]. Thus, our interpretation currently favors that the interaction of Matrin 3 and Rev specifically requires the presence of a Rev-dependent RRE-containing RNA (p37-RRE), but not a Rev-independent RRE-containing RNA (p37-M1-10-RRE). In our experiments, the p37 protein expression levels are similar between p37-RRE, p37-CTE, (Figure 3B, lanes 8-9) and p37M1-10, p37M1-10-RRE and p37-M1-10-CTE (Figure 3B, lanes 10-12); hence, the Matrin 3 - Rev interaction is not influenced by the amount of p37 protein.

### Matrin 3 RNA recognition motifs (RRM) 3 are required for activity on Rev/RRE

**Figure 4:**
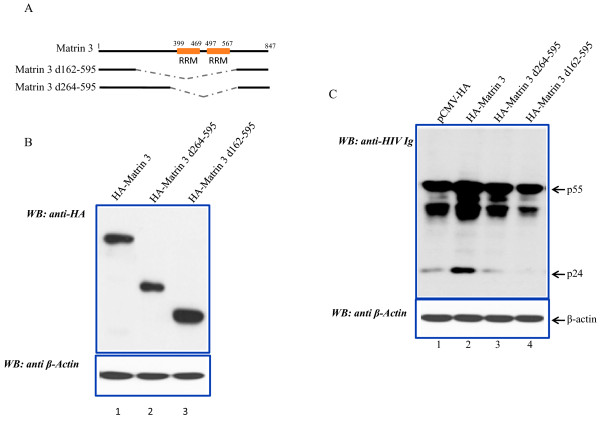
**Matrin 3 RRMs are required for activity on HIV-1 RNA**. A) Schematic representations of the RRM deletion mutants of Matrin 3. B) Western blot verification of the comparable expression of transfected Matrin 3 deletion mutants. Loadings were normalized to β-actin (bottom panel). C) Expression of wild type HA-Matrin 3 (lane 2), but not HA-Matrin 3 d264-595 (lane 3) nor HA-Matrin 3 d162-595 (lane 4), which lack the RRMs activated HIV-1 gene expression as measured by viral p55 or p24 levels.

The above results are consistent with Matrin 3 associating with Rev and RRE-RNA to facilitate expression. A prediction from these results is that an RNA-binding competent Matrin 3 is needed for its activity on HIV-1 RNAs. To address this notion, we constructed two Matrin 3 deletion mutants as indicated in Figure 4A. Matrin 3 is an 847-amino acid protein with two RNA recognition motifs (RRM) contained in amino acids 399 to 567, and a bipartite NLS in amino acids 586 - 612. The RRMs are required for Matrin 3 to bind RNA. The two Matrin 3 deletion mutants expressed well in human cells (Figure 4B). When both were assayed in co-transfections with pNL4-3 (Figure 4C) and compared to the activity of wild type Matrin 3, neither mutant was proficient in activating HIV-1 as measured by Gag p24 expression (Figure 4C). The mutants showed expected localization in the nucleus (Additional file 2 Figure S2). The results from the RRM mutants are consistent with the notion that RNA-binding by Matrin 3 is required for its HIV-1 function.

### Matrin 3 increases the stability and nuclear export of HIV-1 RRE-containing transcripts

**Figure 5:**
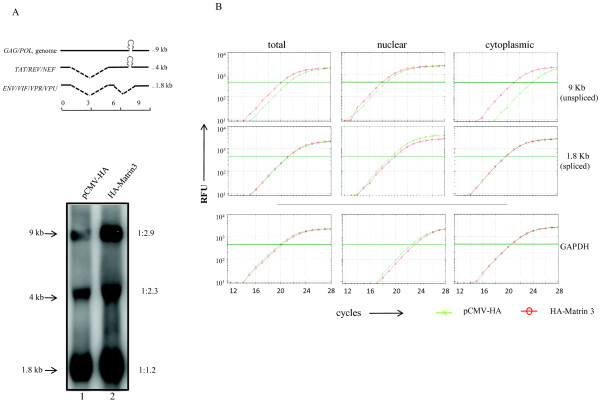
**Matrin 3 stabilizes RRE-containing RNA**. A) (top) Schematic representations of the differently sized mRNA transcripts produced during HIV-1 replication. The 9 kb (unspliced) and 4 kb (singly spliced) viral transcripts contain the RRE cis-element and require Rev protein for expression. (bottom) HeLa cells were transfected with HIV-1 molecular clone pNL4-3 and either pCMV-HA or HA-Matrin 3 plasmids. Northern blot analysis of whole cell RNA demonstrated increased expression of unspliced 9 kb HIV-1 transcript (lane 2). Relative changes in the expression of 9 kb and 1.8 kb HIV-1 RNAs in cells, with and without Matrin 3, are shown by the numbers on the right. B) Matrin 3 increased the stability and promoted the nuclear export of HIV-1 unspliced RNA. HeLa cells were transfected with pNL4-3 with (red) or without (green) Matrin 3. RNA was isolated from whole cell lysates as well as nuclear and cytoplasmic fractions. qRT-PCR analysis of HIV-1 RNA was performed using primers specific for spliced and unspliced viral transcripts [29]. Transfection of Matrin 3 (red) resulted in modestly increased amounts of HIV-1 unspliced transcripts in the cells (top left panel, total), and a much larger increase in the distribution of unspliced HIV-1 transcripts into cytoplasm (top right panel, cytoplasmic). As control, Matrin 3 did not affect the stability or the distribution of GAPDH mRNA (bottom panels, GAPDH).

One consequence of Matrin 3 binding to RNA could be the stabilization of RRE-containing transcript. To check this possibility, we compared the expression of RRE containing transcripts in HeLa cells transfected with HA-Matrin 3 (Figure 5). In HIV-1, the unspliced, partially spliced and fully spliced RNAs can be categorized into three groups based on their sizes. The ~ 9 kb unspliced RNA serves as the genomic RNA and also encodes the Gag, Gag-Pol fusion proteins. A set of ~ 4 kb, singly spliced mRNAs encode for Env, Vpr, Vif and Vpu. A group of fully spliced ~ 1.8 kb mRNAs encode Tat, Rev and Nef. The 9 kb and 4 kb classes of mRNAs contain the RRE element while the 1.8 kb mRNAs do not. We analyzed the effect of Matrin 3 on the expression of the 9 kb and 4 kb transcripts compared to the Rev/RRE independent 1.8 kb group of RNA. HeLa cells were transfected with pNL4-3 and Matrin 3 for this analysis, and we analyzed 20 μg of total RNA by Northern blotting (Figure 5A). There was an increase, in the HA-Matrin 3 transfected cells, in the 9 kb unspliced and 4 kb singly-spliced RNA transcripts (which contain RRE; ratios of 1:2.9 and 1:2.3 respectively; Figure 5A, bottom), compared to the fully spliced 1.8 kb RNA (which does not contain RRE; a ratio of 1:1.2; Figure 5A, bottom).

We next investigated the consequence of increased Matrin 3 expression on cytoplasmic distribution of unspliced versus spliced viral RNAs. We co-transfected HeLa cells with pNL4-3 and Matrin 3, and fractionated cellular RNAs into total, cytoplasmic, or nuclear constituents. We isolated the RNAs from these fractions and analyzed them by qRT-PCR for the levels of unspliced and spliced RNAs using primers specific for the 9 kb or the 1.8 kb viral RNA. We used GAPDH as a normalization control for our fractionation (GAPDH; Figure 5B). Consistent with the Northern blot results, there was a 3 fold increase in expression of unspliced viral RNA in the cells (total 9 kb; Figure 5B), but interestingly the amount of 9 kb viral RNA distributed into the cytoplasm of pCMV-HA-Matrin 3 expressing cells was 10 fold higher than that found in pCMV-HA expressing cells (cytoplasmic 9 kb; Figure 5B; also see Additional file 3, Figure S3). By contrast, the distribution and expression of spliced RNA remained unchanged in the presence of increased Matrin 3 expression (1.8 kb; Figure 5B). These results are consistent with the interpretation that Matrin 3 can selectively stabilize and increase the nuclear to cytoplasmic distribution of unspliced 9 kb vs. spliced 1.8 kb HIV-1 RNAs.

## Discussion

Here, we have shown that nuclear matrix protein Matrin 3 influences the expression of HIV-1 RRE-containing mRNAs. Matrin 3 acts post-transcriptionally via Rev/RRE to increase the expression of HIV-1 Rev/RRE dependent unspliced or partially spliced transcripts. This activity requires Matrin 3 to bind Rev-dependent RRE-containing RNA and appears to lead to the stabilization and nuclear to cytoplasmic export of RRE-containing HIV-1 transcripts.

Previously it was shown that Matrin 3 exists in cells complexed with PSF (PTBP associated splicing factor) and nrbp54 [53,65-67]. Others have found that PSF binds to instability elements (INS) contained within the HIV-1 transcripts and suppresses the expression of these RNAs [59]. The INS elements are primarily present in the RRE-containing unspliced and partially spliced viral transcripts [31,64,68-72]. It is possible that some of the effects that we have observed from Matrin 3 may be due to its interaction with PSF and p54nrb. That Matrin 3 might counter the reported PSF-suppression of RNA expression has not been explored here, but it remains important to establish and clarify this mechanistic interaction in the future.

Our results are compatible with a model in which Matrin 3 binds to RRE containing transcripts and stabilizes them in the presence of Rev, which then directs these viral transcripts for export out of the nucleus. This interpretation is supported by our observation that Rev - Matrin 3 interaction is RRE-RNA dependent, and Matrin 3 activity requires the presence of Rev and RRE-containing RNA. Further experiments are needed to answer the mechanistic details of how Matrin 3 and Rev cooperate in their interactions with RRE-containing RNA. One intriguing finding is that Matrin 3 has been identified as a constituent of the nuclear pore proteomes [73]; this localization would be compatible with Matrin 3 being a part of an RNP-complex that exits the nucleus into the cytoplasm through the nuclear pore. Also of interest, Bushman *et al*. [74] recently performed a meta-analysis of published genome-wide siRNA screening of cellular factors important for HIV-1 replication. They used a graph theory clustering algorithm (MCODE) to assemble a HIV-1 host interactome in which nuclear matrix structure (Matrin 3) was identified as an interactor with the molecular chaperone cluster identified by siRNA-screening as involved in the assembly of viral proteins. Our evidence here for a role of Matrin 3 in HIV-1 post-transcriptional RNA expression is consistent with the above analysis. In conclusion, the implication of Matrin 3 as an additional Rev co-factor adds further complexity to the understanding of post-transcriptional regulation of unspliced/partially spliced HIV-1 RNA. Although it remains to be established, Matrin 3 may be a cellular factor that counters the nuclear retention through INS elements of HIV-1 unspliced/partially spliced RNAs.

## Materials and methods

### Plasmids

Full-length Matrin 3 clone was purchased from Open Biosystems and cloned into pCMV-HA vector (Clontech) by PCR. HIV-1 LTR luciferase plasmid, pCMV-NL-GagPol-RRE and pCMV-NL-GagPol-CTE were from E. Freed and D. Rekosh. Plasmids p37 and p37RRE were kindly provided by B. Felber [64] and cloned into pcDNA3.

### Cell Culture, Transfection, and Reporter Assays

Cell propagation, transfection, qRT-PCR and reporter assays were as described previously [28,29]. All transfections were repeated three or more times and were normalized to β-galactosidase activity expressed from a co-transfected pCMV-β (Clontech).

### Antibodies

Mouse monoclonal anti-HA (Sigma Chemical); mouse monoclonal Matrin 3, (Abcam) and rabbit anti-GFP and anti-HA (Cell Sciences) are commercially available.

### Western Blotting, and Immunoprecipitation

Western blotting and immunoprecipitation were performed as described previously [28,29]. Briefly, the cells were washed twice with PBS and lysed with sample buffer [100 mMTris (pH6.8), 4%SDS, 20% glycerol, 5% β-mercaptoethanol, and 0.05% bromophenol blue]. Cell lysates were boiled for 10 minutes, and loaded onto a SDS/PAGE gel and electrophoresed. The gel was electroblotted onto Immobilon-P membranes (Millipore) and probed with the primary antibodies, followed by incubation with anti-rabbit, anti-mouse, or anti-human alkaline phosphatase-conjugated secondary antibody and detected using a chemiluminescence substrate (Applied Biosystems).

### RNA isolation, Northern blotting and qRT-PCR

Total RNA from cells was extracted with Tri-Reagent (Sigma-Aldrich). Nuclear and cytoplasmic RNAs were isolated by cell fractionation (Paris Kit; Applied Biosystems), and RNA was isolated with Tri-Reagent. Northern blots were performed as described previously [28]. Extracted RNA was analyzed by qRT-PCR using the iScript One-Step RT-PCR Kit with SYBR Green (Bio-Rad) according to manufacturer's instructions. Samples were reverse-transcribed at 50°C for 30 minutes, and amplification was performed after an initial step at 95°C for 10 minutes, followed by 20-40 cycles at 95°C for 30 s, 55°C for 30 s, and 72°C for 60 s. The primers and their sequences used in the analyses have been previously described [29]. Primers for unspliced transcripts were Primer A 5′-GTCTCTCTGGTTAGACCAG-3′, Primer C 5′-CTAGTCAAAATTTTTGGCGTACTC-3′ and primer A and sj4.7A 5′- TTGGGAGGTGGGTTGCTTTGATAGAG-3 for spliced 2 Kb transcript. For GAPDH forward 5′ CTCTGCTCCTCCTGTTCGAC 3′ and GAPDH reverse 5′ TTAAAAGCAGCCCTGGTGAC 3′ primers were used.

### Co-immunoprecipitation

Co-immunoprecipitation assay has been described previously [28,29]. Cell lysates were prepared in RIPA buffer [Tris-buffered saline (pH 8.0) containing 1% Triton X-100 or Nonidet P-40, 1 mg of BSA/mL, and 1 mM EDTA] containing (phenylmethylsulfonyl fluoride and aprotinin 10 μg/mL), 0.5% sodium deoxycholate, and 0.1% SDS. Cell lysates were prepared and incubated at 4°C overnight with the indicated antibodies and immune complexes were pulled down using protein G-agarose beads and analyzed by Western blotting.

## Competing interests

The authors declare that they have no competing interests.

## Authors' contributions

VSY performed all the experiments. VSY and KTJ designed the experiments and wrote the manuscript. Both authors read and approved the final manuscript.

## Supplementary Material

Additional file 1 **Figure S1. Overexpression of Matrin 3 rescues Matrin3 siRNA mediated suppression of HIV-1 gene expression**. HeLa cells were transfected with Matrin 3 siRNA along with pNL4-3 and the indicated Matrin3 expression constructs. Cell lysates were collected and analyzed by Western blotting. As shown the Matrin3 siRNA knocked down cell endogenous Matrin3 (compare lane 1 and 2, middle panel), but the overexpression of Matrin3 restored the Matrin3 levels in the cell (compare lane 1 and 6 middle panel). Knockdown of Matrin3 suppressed HIV-1 gene expression as indicated by measured p24 levels (lane 2); conversely the increased expression of Matrin3 from transfected plasmids restored HIV-1 gene expression (lane 6). Click here for file

Additional file 2 **Figure S2. Matrin 3 deletion mutants localize to the nucleus**. HeLa cells were transfected with the indicated Matrin 3 deletion mutants; cells were fixed and stained with anti-HA antibody and alexa 488 tagged secondary antibody. Intracellular distribution of matrin3 was examined by confocal imaging. Click here for file

Additional file 3 **Figure S3. Matrin 3 increased the stability and promoted the nuclear export of HIV-1 unspliced RNA**. The experiment in Figure 5B was repeated in triplicate, and qRT-PCR results from two representative repeats are presented here. HeLa cells were transfected with pNL4-3 along with (red) or without (green) Matrin 3. RNA was isolated from whole cell lysates as well as nuclear and cytoplasmic fractions. qRT-PCR analysis of HIV-1 RNA was performed using primers specific for spliced and unspliced viral transcripts. Transfection of Matrin 3 (red) resulted in modestly increased amounts of HIV-1 unspliced transcripts in the cells (top left panels, total), and a much larger increase in the distribution of unspliced HIV-1 transcripts into the cytoplasm (top right panels, cytoplasmic). As control, Matrin 3 did not affect the stability or the distribution of GAPDH mRNA (bottom panels, GAPDH). RFU = relative fluorescent units. Click here for file
