# Supplementary material for: Matrin 3 is a co-factor for HIV-1 Rev in regulating post-transcriptional viral gene expression
Source: Retrovirology. 2011 Jul 20;8:61. doi: 10.1186/1742-4690-8-61 (PMC3160905; doi:10.1186/1742-4690-8-61)
Supplement: Additional file 1 — Figure S1. Overexpression of Matrin 3 rescues Matrin3 siRNA mediated suppression of HIV-1 gene expression. HeLa cells were transfected with Matrin 3 siRNA along with pNL4-3 and the indicated Matrin3 expression constructs. Cell lysates were collected and analyzed by Western blotting. As shown the Matrin3 siRNA knocked down cell endogenous Matrin3 (compare lane 1 and 2, middle panel), but the overexpression of Matrin3 restored the Matrin3 levels in the cell (compare lane 1 and 6 middle panel). Knockdown of Matrin3 suppressed HIV-1 gene expression as indicated by measured p24 levels (lane 2); conversely the increased expression of Matrin3 from transfected plasmids restored HIV-1 gene expression (lane 6). [file 1742-4690-8-61-S1.PDF]

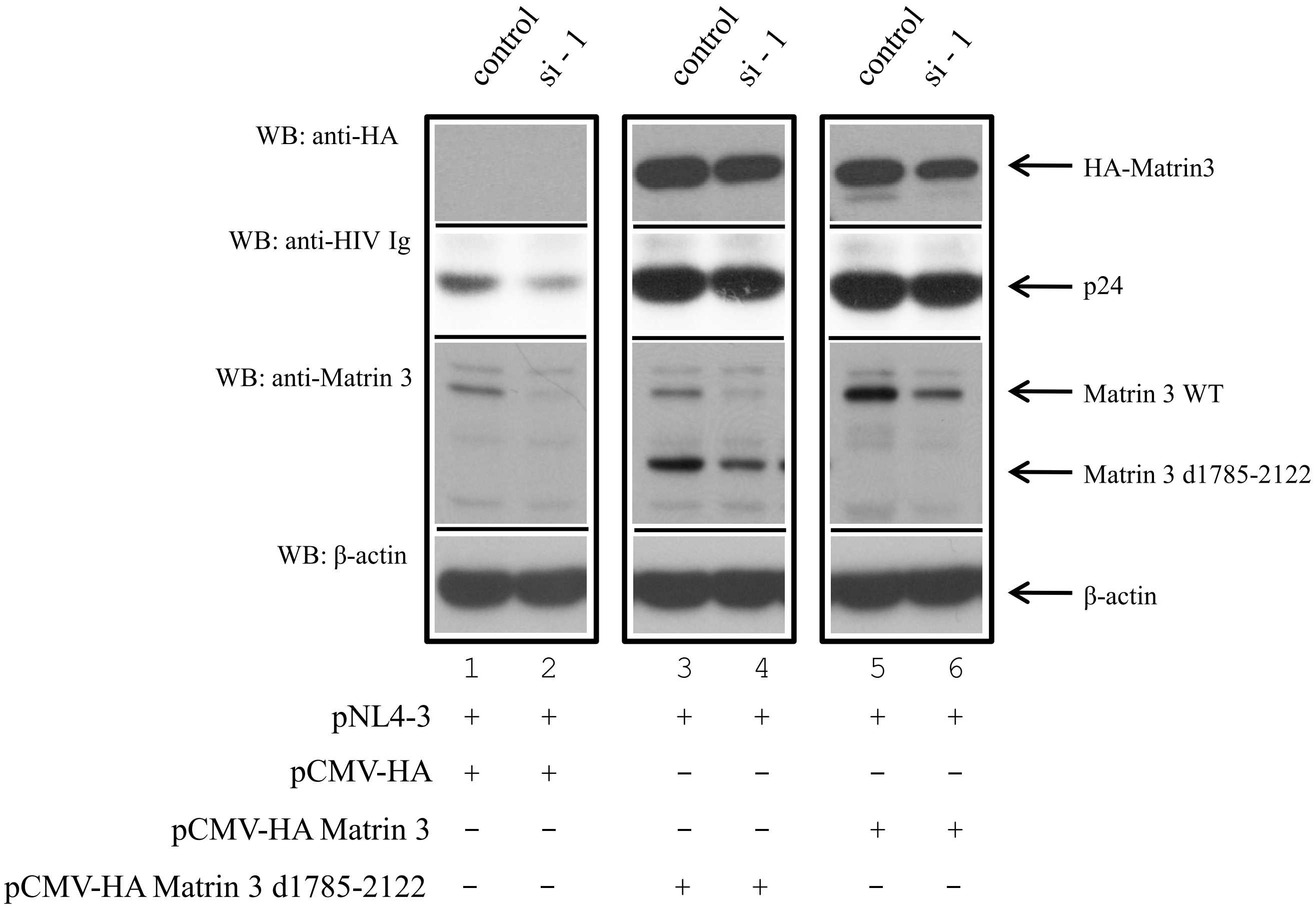

Supplemental figure 1. Overexpression of Matrin 3 rescues matrin 3 siRNA mediated suppression of HIV-1 gene expression. HeLa cells were transfected with Matrin 3 siRNA along with pNL4-3 and the indicated matrin 3 expression constructs. Cell lysates were collected and analyzed by Western blotting. As shown the matrin 3 siRNA knocked down cell endogenous matrin 3 (compare lane 1 and 2, middle panel), but the overexpression of matrin 3 restored the the matrin 3 levels in the cell (compare lane 1 and 6 middle panel). Knockdown of matrin 3 suppressed HIV-1 gene expression as indicated by measured p24 levels (lane 2); conversely the increased expression of matrin 3 from transfected plasmids restored HIV-1 gene expression (lane 6).
