# Supplementary material for: Matrin 3 is a co-factor for HIV-1 Rev in regulating post-transcriptional viral gene expression
Source: Retrovirology. 2011 Jul 20;8:61. doi: 10.1186/1742-4690-8-61 (PMC3160905; doi:10.1186/1742-4690-8-61)
Supplement: Additional file 2 — Figure S2. Matrin 3 deletion mutants localize to the nucleus. HeLa cells were transfected with the indicated Matrin 3 deletion mutants; cells were fixed and stained with anti-HA antibody and alexa 488 tagged secondary antibody. Intracellular distribution of matrin3 was examined by confocal imaging. [file 1742-4690-8-61-S2.PDF]

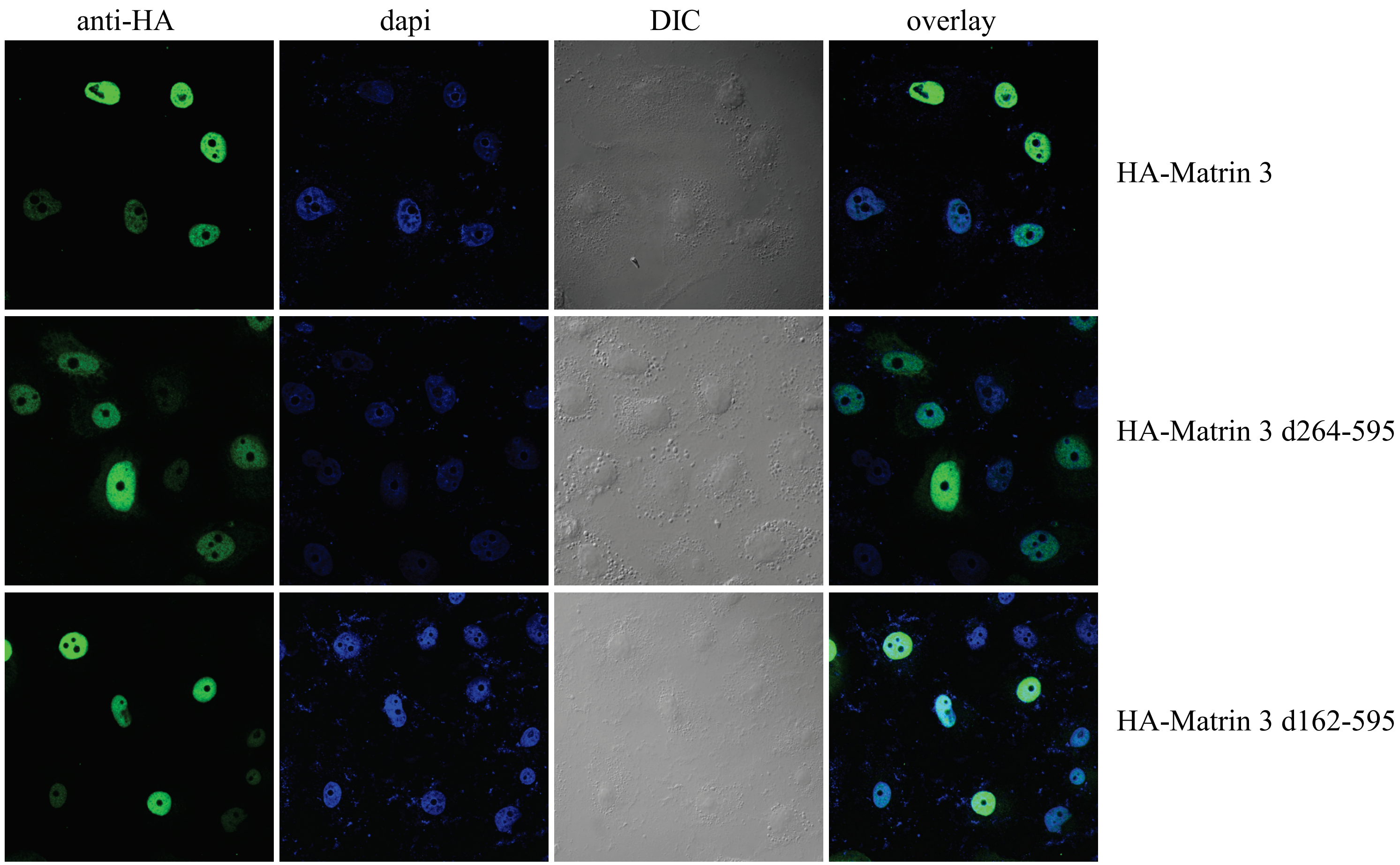


**Supplemental figure 2. Matrin 3 deletion mutants localize to the nucleus.** HeLa cells were transfected with the indicated Matrin 3 deletion mutants, cells were fixed and stained with anti-HA antibody and alexa 488 tagged secondary antibody. Intracellular distribution of matrin 3 was examined by confocal imaging.
