# Supplementary material for: Matrin 3 is a co-factor for HIV-1 Rev in regulating post-transcriptional viral gene expression
Source: Retrovirology. 2011 Jul 20;8:61. doi: 10.1186/1742-4690-8-61 (PMC3160905; doi:10.1186/1742-4690-8-61)
Supplement: Additional file 3 — Figure S3. Matrin 3 increased the stability and promoted the nuclear export of HIV-1 unspliced RNA. The experiment in Figure 5B was repeated in triplicate, and qRT-PCR results from two representative repeats are presented here. HeLa cells were transfected with pNL4-3 along with (red) or without (green) Matrin 3. RNA was isolated from whole cell lysates as well as nuclear and cytoplasmic fractions. qRT-PCR analysis of HIV-1 RNA was performed using primers specific for spliced and unspliced viral transcripts. Transfection of Matrin 3 (red) resulted in modestly increased amounts of HIV-1 unspliced transcripts in the cells (top left panels, total), and a much larger increase in the distribution of unspliced HIV-1 transcripts into the cytoplasm (top right panels, cytoplasmic). As control, Matrin 3 did not affect the stability or the distribution of GAPDH mRNA (bottom panels, GAPDH). RFU = relative fluorescent units. [file 1742-4690-8-61-S3.PDF]

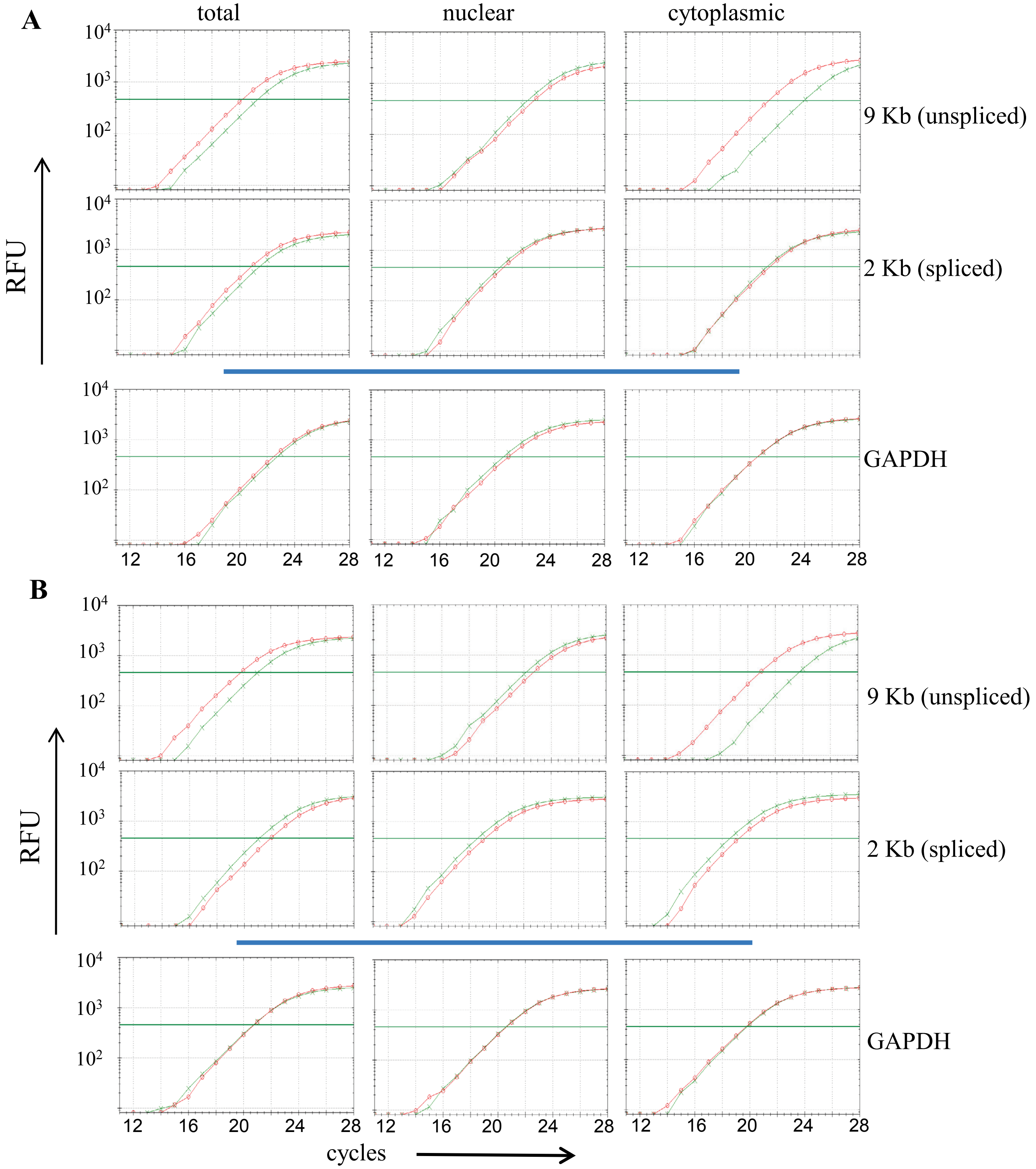

Supplemental figure 3: Matrin 3 increased the stability and promoted the nuclear export of HIV-1 unspliced RNA. The experiment in Figure 5B was repeated in triplicate and qRT-PCR results from two representative repeats are presented here. HeLa cells were transfected with pNL4-3 along with (red) or without (green) Matrin 3. RNA was isolated from whole cell lysates as well as nuclear and cytoplasmic fractions. qRT-PCR analysis of HIV-1 RNA was performed using primers specific for spliced and unspliced viral transcripts. Transfection of Matrin 3 (red) resulted in modestly increased amounts of HIV-1 unspliced transcripts in the cells (top left panels, total), and a much larger increase in the distribution of unspliced HIV-1 transcripts into cytoplasm (top right panels, cytoplasmic). As control, Matrin 3 did not affect the stability or the distribution of GAPDH mRNA (bottom panels, GAPDH). RFU = relative fluorescent units.
